# Artificial Intelligence in the Management of Infectious Diseases in Older Adults: Diagnostic, Prognostic, and Therapeutic Applications

**DOI:** 10.3390/biomedicines13102525

**Published:** 2025-10-16

**Authors:** Antonio Pinto, Flavia Pennisi, Stefano Odelli, Emanuele De Ponti, Nicola Veronese, Carlo Signorelli, Vincenzo Baldo, Vincenza Gianfredi

**Affiliations:** 1 Faculty of Medicine, University Vita-Salute San Raffaele, 20132 Milan, Italy; pinto.antonio@hsr.it (A.P.);; 2 PhD National Program in One Health Approaches to Infectious Diseases and Life Science Research, Department of Public Health, Experimental and Forensic Medicine, University of Pavia, 27100 Pavia, Italy; 3 Faculty of Medicine, Saint Camillus International University of Health Sciences, 00131 Rome, Italy; 4 Department of Cardiac Thoracic Vascular Sciences and Public Health, University of Padua, 35100 Padova, Italy

**Keywords:** artificial intelligence, machine learning, geriatric infections, older adults, infectious diseases, diagnostic algorithms, prognostic models, geriatric medicine, sepsis prediction, antimicrobial stewardship

## Abstract

**Background**: Older adults are highly vulnerable to infectious diseases due to immunosenescence, multimorbidity, and atypical presentations. Artificial intelligence (AI) offers promising opportunities to improve diagnosis, prognosis, treatment, and continuity of care in this population. This review summarizes current applications of AI in the management of infections in older adults across diagnostic, prognostic, therapeutic, and preventive domains. **Methods**: We conducted a narrative review of peer-reviewed studies retrieved from PubMed, Scopus, and Web of Science, focusing on AI-based tools for infection diagnosis, risk prediction, antimicrobial stewardship, prevention of healthcare-associated infections, and post-discharge care in individuals aged ≥65 years. **Results**: AI models, including machine learning, deep learning, and natural language processing techniques, have demonstrated high performance in detecting infections such as sepsis, pneumonia, and healthcare-associated infections (Area Under the Curve AUC up to 0.98). Prognostic algorithms integrating frailty and functional status enhance the prediction of mortality, complications, and readmission. AI-driven clinical decision support systems contribute to optimized antimicrobial therapy and timely interventions, while remote monitoring and telemedicine applications support safer hospital-to-home transitions and reduced 30-day readmissions. However, the implementation of these technologies is limited by the underrepresentation of frail older adults in training datasets, lack of real-world validation in geriatric settings, and the insufficient explainability of many models. Additional barriers include system interoperability issues and variable digital infrastructure, particularly in long-term care and community settings. **Conclusions**: AI has strong potential to support predictive and personalized infection management in older adults. Future research should focus on developing geriatric-specific, interpretable models, improving system integration, and fostering interdisciplinary collaboration to ensure safe and equitable implementation.

## 1. Introduction

As populations age globally, the demographic shift brings a heavier burden of infectious disease among older adults. Although many communicable diseases have declined in younger groups, older people remain disproportionately vulnerable or frail. Age-associated immune decline, known as immunosenescence and inflammaging, reduces adaptive immunity and increases baseline systemic inflammation, thereby impairing pathogen response and worsening infection outcomes [1,2,3]. Between 2010 and 2024, hospital admissions of older patients rose nearly 25%, reflecting rising age-related illnesses and infectious complications among older people [4].

Forecast models estimate that by 2050, the prevalence of chronic diseases and disability among older adults will rise sharply, with related disability nearly doubling [5]. Thus, the intersection of population ageing and infectious risk is clear: older adults face a higher incidence of infections (e.g., pneumonia, influenza, SARS-CoV-2), a worse mortality rate, and more prolonged or disabling recovery trajectories [6,7]. Furthermore, older adults are particularly vulnerable not only to infectious diseases but also to indirect health threats exacerbated by environmental and social stressors, such as climate-related disruptions to food systems and nutrition [8].

The clinical complexity of older patients presents multiple intertwined challenges. Multimorbidity, often defined as the concomitant presence of 2 or more chronic physical conditions, involving several organ systems rather than isolated conditions, is extremely prevalent: studies report 55% to 98% of older adults presenting with complex multimorbidity, linked to higher mortality and long-term care needs [9,10,11]. Common conditions include hypertension, diabetes, heart disease, chronic lung disease, and sarcopenia, the latter affecting 11–50% of those over 80, and strongly associated with falls, disability, infection vulnerability, and mortality [12].

Additionally, older patients often present atypically. Instead of textbook symptoms, infections may manifest as confusion, falls, delirium or functional decline, leading to delayed diagnosis and worse outcomes [13]. Underlying frailty, cognitive impairment, and sensory deficits, using a comprehensive geriatric assessment approach [14], compound this clinical ambiguity.

Polypharmacy is another major concern: many older patients use multiple medications for comorbid conditions, increasing risks of drug–drug interactions, adverse effects, and reduced physiological reserve. Decisions about stopping potentially inappropriate medications require nuanced, personalized risk-benefit analyses, especially when medications offer marginal benefit or harm in advanced age or multimorbidity [15].

All these factors combine to make geriatric clinical care highly complex: the same presenting sign may mask multiple underlying conditions; standard protocols may be ill-suited for frail, multimorbid individuals, and routine lab or imaging interpretations may be clouded by age-related changes [16]. Recent clinical experimental studies increasingly highlight the need for new models of geriatric assessment that account for multimorbidity, frailty, and medication load together.

**Figure 1:**
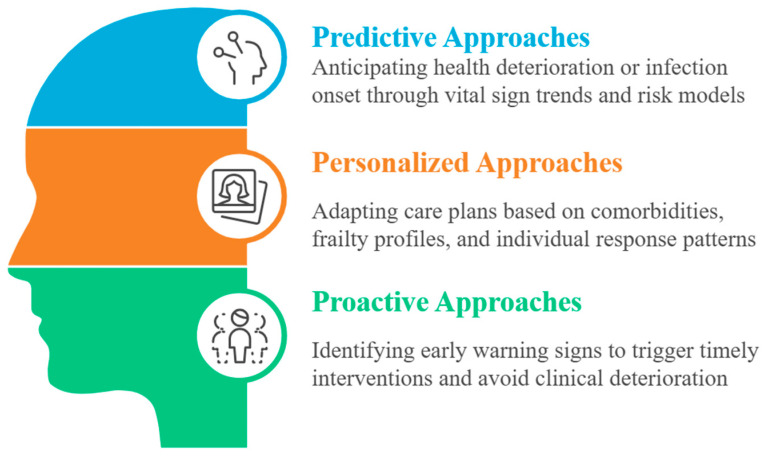
Overview of three of the main roles of artificial intelligence in geriatric care.

In this regard, Artificial Intelligence (AI) may offer powerful tools to reshape geriatric medicine along three axes: predictive, personalized, and proactive (Figure 1) [17].

For example, Machine Learning (ML) models, including deep learning and time-series analysis, can analyse longitudinal datasets and wearable sensor signals to anticipate adverse events. A typical example is the prediction of dementia in people initially free from this condition [18]. Another broad review of AI in remote patient monitoring (2023) demonstrated the capacity to detect early deterioration in chronic and acute illness via continuous analysis of vital signs and behaviours, using federated learning to preserve privacy and personalize thresholds [19]. Furthermore, AI methods are already being applied to customize care for older adults. Surveys and clinical trials show older patients respond well to AI-based medication advice systems, though they value transparency and trust in the source [20]. A recent review highlights applications in fall prediction, cognitive decline tracking, and multi-morbidity management, using integrated patient data, genomics, physiology, and environment to tailor risk assessments and treatment strategies [21].

Finally, about the topic of proactive care in geriatrics, emerging work on agentic AI, often Large Language Model (LLM)-powered, points toward systems capable of autonomous and pre-emptive decision support. A 2025 theoretical study described AI agents that monitor health, environment, cognition, and daily routines, anticipating declines or hazards and intervening (e.g., suggesting hydration, scheduling check-ins, alerting clinicians), all while preserving autonomy and privacy [22]. Complementary research in nursing perspectives (2024) emphasized AI’s role in assisting diagnoses, continuous monitoring, and personalized alerts, enabling a proactive rather than reactive model of geriatric care [23]. Finally, an important 2025 review explicitly addressed the intersection of AI and precision geriatric medicine, citing ongoing trials and pilot studies using predictive analytics to stratify patients, forecast adverse drug effects, tailor therapy plans, and support clinical decision-making in older adult populations [24]. These systems may integrate genomic, behavioural, physiological, and pharmacological data to optimize interventions and reduce burdens of comorbidity and polypharmacy.

## 2. Methods

This study followed a narrative review design aimed at synthesizing current evidence on AI applications for the management of infectious diseases in older adults. We searched three major electronic databases, PubMed, Scopus, and Web of Science, for peer-reviewed articles published up to July 2025. The search strategy combined keywords and MeSH terms related to AI, machine learning, deep learning, natural language processing, infectious diseases, older adults, geriatrics, diagnosis, prognosis, antimicrobial stewardship, healthcare-associated infections, and post-discharge care.

We included original studies, systematic reviews, and meta-analyses that reported on the use of AI- or machine learning-based tools in individuals aged ≥65 years, or that provided geriatric-specific analyses. Studies were eligible if they addressed one or more of the following areas: (1) diagnosis of infectious diseases, (2) risk stratification or prognostic modelling, (3) optimization of antimicrobial therapy, (4) prevention and surveillance of healthcare-associated infections (HAIs), or (5) continuity of care after hospital discharge. Articles not available in English, editorials, commentaries, and studies not involving older adult populations were excluded. Titles and abstracts retrieved through the search were screened for relevance, and full texts were reviewed when eligibility was uncertain. Studies meeting the inclusion criteria were synthesized qualitatively and organized by thematic domain (diagnosis, prognosis, antimicrobial stewardship, prevention of HAIs, and continuity of care).

Relevant information was categorized according to clinical application areas, AI methodologies used, reported outcomes (e.g., diagnostic accuracy, AUC, sensitivity), and any geriatric-specific adaptations. The results were synthesized qualitatively and presented across thematic domains.

## 3. Artificial Intelligence Algorithms Applicable to the Diagnosis of Infections in Older People

**Figure 2:**
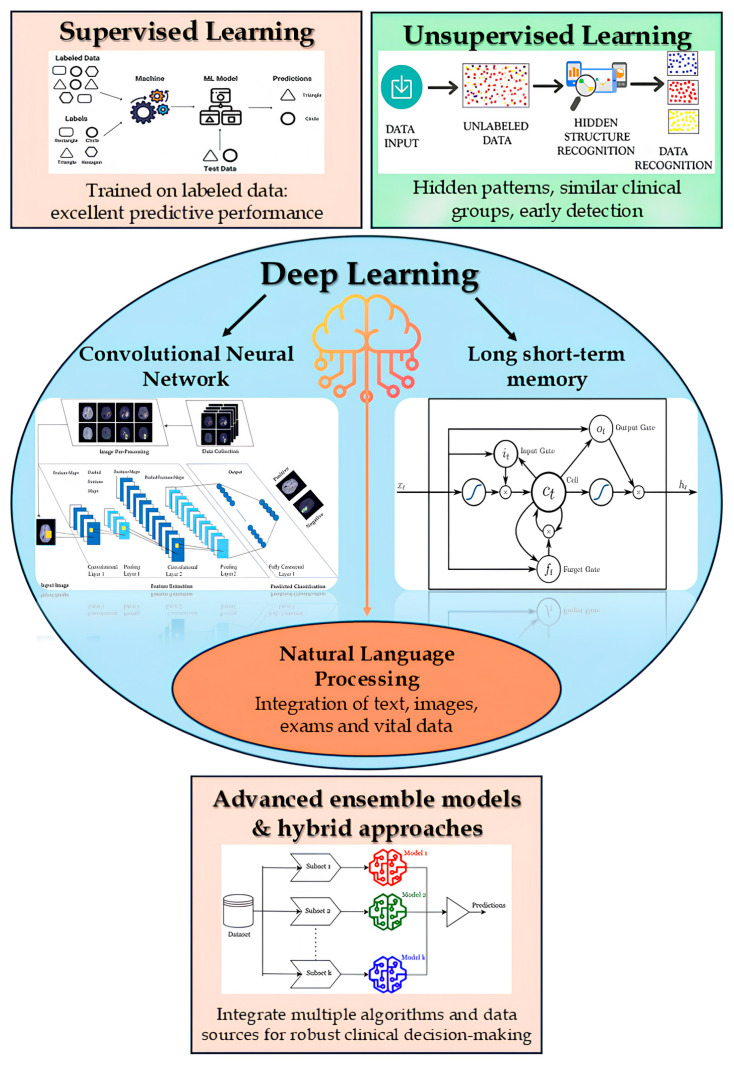
Overview of artificial intelligence methodologies applicable to infection diagnosis in older adults. ML: Machine Learning.

AI methodologies applicable to the diagnosis of infections in older adults encompass a wide spectrum of approaches, ranging from supervised and unsupervised learning to deep learning and natural language processing (NLP) (Figure 2) [25].

Within the realm of supervised learning, classical algorithms, such as logistic regression, Support Vector Machines (SVM), and k-nearest neighbors, are trained on labelled clinical datasets to enable early classification of infectious episodes or to estimate individualized risk profiles. In particular, ensemble methods, including Random Forests and boosting-based models such as XGBoost, frequently yield superior performance by integrating the predictive capabilities of multiple decision trees or classifiers [26]. For instance, Random Forest has achieved an area under the curve (AUC) of approximately predicting nosocomial infections from surveillance data [27], while an XGBoost-based model reached an AUROC of ~0.97 in the early detection of sepsis among elderly patients [28].

In contrast, unsupervised learning techniques, such as k-means clustering and anomaly detection models, do not require labeled data and are instrumental in uncovering latent patterns or grouping patients with similar clinical profiles. These methods can aid in the early identification of atypical configurations in vital signs or laboratory results suggestive of infection [29].

Deep learning further enhances diagnostic capabilities using multilayer neural networks [30]. Convolutional Neural Networks (CNNs), for example, autonomously extract salient features from complex inputs such as radiological images, enabling the identification of pneumonia on chest X-rays with a diagnostic accuracy comparable to that of experienced radiologists [31]. Similarly, recurrent neural architectures such as Long Short-Term Memory (LSTM) networks and contemporary Transformer-based models are adept at capturing temporal dependencies within clinical monitoring data. These networks can analyze sequential trends in physiological parameters (e.g., vital signs, laboratory trajectories) to forecast severe infectious events, such as the onset of sepsis, well in advance of clinical manifestation [32]. Recent studies have demonstrated that Transformer-based models applied to time-series data of vital signs can achieve predictive accuracies of approximately 96% up to 12 h before sepsis onset [33].

Concurrently, Natural Language Processing (NLP) facilitates the analysis of unstructured clinical texts. Neural models pre-trained on biomedical corpora, such as BERT-based Transformers, can extract infection-related indicators from medical reports, clinical notes, and nursing documentation, often capturing symptomatology and signs of infection overlooked by systems relying solely on structured data [34]. The integration of NLP techniques with traditional electronic health record (EHR) data has been shown to enhance infection surveillance: for example, the extraction of relevant information from narrative notes via NLP, when combined with structured EHR variables, has improved both specificity and predictive accuracy in the monitoring of surgical site infections [35].

Finally, advanced ensemble models and hybrid approaches are emerging as effective strategies for multimodal data integration in the clinical domain. Decision-support systems that synthesize diverse data sources, such as real-time vital signs, laboratory results, diagnostic imaging, and free-text documentation, through complementary algorithmic components offer a robust diagnostic framework by leveraging the strengths of multiple methodologies [36]. These hybrid strategies, such as neural networks capable of fusing heterogeneous data or ensembles that aggregate outputs from diverse models, are explained to enhance the accuracy and reliability of early diagnosis, thereby enabling the timely identification of infections in older adults and guiding proactive clinical interventions [37].

## 4. Early Diagnosis and Recognition of Atypical Presentations

**Figure 3:**
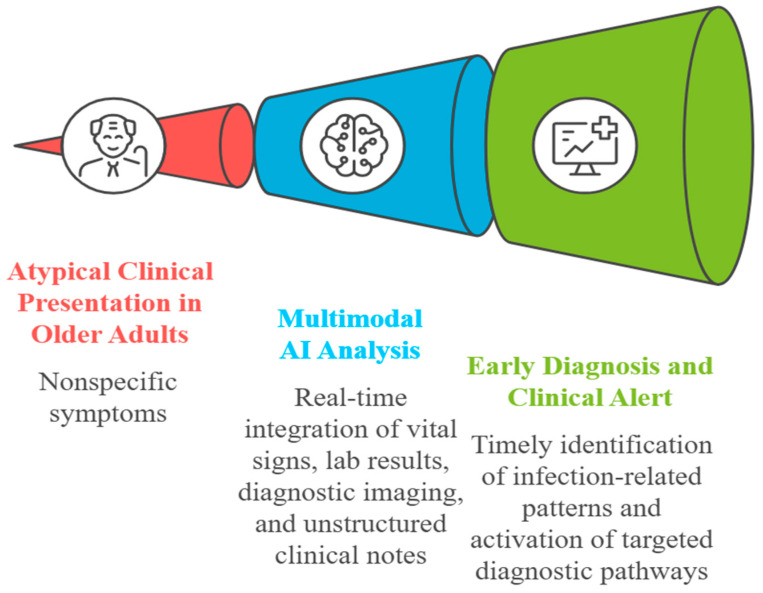
Clinical-pathway view: atypical presentations at triage feed multimodal artificial intelligence (AI) (vitals, labs, imaging, notes) to generate early diagnostic alerts and trigger targeted infection work-ups.

AI algorithms represent a strategic asset for the early diagnosis of infections in older adults, owing to their capacity to integrate and simultaneously analyze heterogeneous clinical data from multiple sources. The real-time combination of vital signs, laboratory results, diagnostic imaging, and unstructured text from EHRs enables the overcoming of limitations inherent to traditional clinical interpretation, which is often fragmented and subject to subjective bias [38,39]. In particular, infectious risk stratification in clinically complex patients benefits from the multimodal analysis provided by AI systems, which are capable of detecting subtle yet clinically meaningful variations in the patient’s condition. This capability is especially valuable in older adults, in whom infections often present with vague or atypical symptoms (Figure 3) [40].

In older adults, infections often manifest through subtle and atypical signs, which may be easily overlooked or misattributed. Algorithms trained on large geriatric datasets can detect these weak signals early and place them within a coherent clinical framework, facilitating the timely initiation of targeted diagnostic pathways.

Numerous studies have highlighted how the implementation of AI-based predictive models can significantly reduce diagnostic delays and contribute to the prevention of inappropriate hospitalizations. For example, an early warning system based on a random forest model, integrated into a university hospital, anticipated the identification of sepsis by over eight hours compared to clinical staff, with a positive impact on the timing of antibiotic therapy and overall clinical outcomes [41]. In geriatric settings as well, the application of predictive models within internal medicine wards has been associated with a reduction in avoidable emergency department visits and improved appropriateness of hospital admissions.

The applications of such tools extend across various care settings. In hospitals, AI algorithms can support triage, continuous surveillance, and personalized infectious risk management; in long-term care facilities, they enable proactive monitoring of residents and early outbreak detection; in home care and telemedicine, integrated sensors and remote predictive models can detect even minimal clinical changes, allowing for intervention before hospitalization becomes necessary [42]. These applications underscore the transformative potential of AI in the management of infections among older adults, promoting a predictive, timely, and person-centered model of care.

## 5. Risk Stratification and Prediction of Clinical Outcomes

The use of AI in risk stratification and prediction of clinical outcomes in older adults with infectious diseases has expanded in recent years, driven by the growing ageing population and the continuous advancements in AI technology [43]. In this context, AI-based models have proven to be valuable tools for estimating critical outcomes such as sepsis [44,45,46,47,48], septic shock [49], mortality [50], and the occurrence of complications [51] in older adults, a population at high risk of adverse events due to comorbidities and increased frailty [52]. In many cases, current diagnostic methods and predictive algorithms lack specificity for this age group. By leveraging large datasets and advanced algorithms, AI models can instead predict the likelihood of adverse events more accurately, and support clinicians in identifying high-risk patients to enable timely interventions.

A meta-analysis by Zhang et al. (2024) [44] showed that ML algorithms have excellent diagnostic accuracy in predicting the occurrence of sepsis, suggesting potential for clinical use. Similarly, Lin et al. (2025) [45] introduced an ML model that utilizes non-invasive routine tests, such as complete blood count data, to accurately predict sepsis in older patients in the intensive care unit (ICU). This model, integrated within an AI-based clinical decision support system (AI-CDSS), enabled early sepsis detection and intervention in critical care settings, improving patient outcomes. Chua et al. (2025) [49] also emphasized AI’s role in managing sepsis within emergency departments, showing its utility in predicting progression to septic shock and mortality. In terms of mortality prediction, Zhang et al. (2025) [50] validated an ML model employing sepsis-predictive algorithms capable of estimating 28-day mortality risk in elderly sepsis patients. The model proved to be both rapid and practical, supporting early risk assessment and prompt clinical decision-making. Recent advancements have further refined AI prediction models, extending their utility to conditions such as multi-organ failure (MOF). For example, Rajakaruna et al. (2024) [51] showed the potential of an AI-driven approach in predicting the onset of MOF in COVID-19 patients. 

Another important aspect of AI’s role in infectious disease management is the ability to personalize prognostic assessments based on factors such as frailty, cognitive status, and functional performance, which significantly impact clinical outcomes in older adults but are often challenging if assessed using traditional methods. A review by Velazquez-Diaz et al. (2023) [53] reported that AI models have been effectively used to identify frailty syndrome using both clinical and non-clinical data, including activity monitoring. Models [54] incorporating frailty indicators have shown the ability to predict adverse outcomes such as prolonged hospitalization, complications, readmissions, and mortality, underlining the promise of AI in delivering individualized care. However, transitioning these tools from research to clinical practice remains a complex challenge. AI indeed shows promise in predicting cognitive decline [55], a key concern in geriatric health, opening opportunities for early intervention. In the context of infectious diseases, this application is particularly important: older sepsis survivors are at a significantly higher risk [56] of long-term cognitive impairment and functional disability. Developing AI models capable of identifying those at risk could enable timely interventions to prevent or mitigate these long-term consequences.

AI-powered clinical decision support systems (CDSS) are increasingly integrated into practice, enhancing decision-making in the management of infectious diseases in older adults. These systems combine patient-specific data to recommend personalized treatment plans. For instance, Düvel et al. (2025) [57] evaluated the feasibility of an AI-based CDSS prototype for guiding antibiotic therapy in sepsis, while Lin et al. (2025) [45] found that such systems improved the management of sepsis in elderly patients by aiding clinicians in assessing treatment timing and appropriate care levels. Moreover [58], AI-based CDSS utilizing EHR data can prioritize treatment alternatives tailored to the complexities of geriatric care. These systems provide clinicians with actionable insights for early, informed decisions about targeted therapies and advanced care planning, helping to align clinical choices with both medical needs and patient preferences.

Beyond individual decision support, AI holds promise for enhancing coordination among multidisciplinary teams, essential in managing complex cases like infectious diseases in older adults. AI models can integrate and synthesize data across various disciplines (geriatrics, infectious diseases, intensive care), offering a comprehensive clinical picture. One study [59] showcased a digital platform powered by AI that supported care coordination and shared care planning in frailty-focused elder care. Such evidence indicates that integrating AI into multidisciplinary care pathways may lead to improved outcomes and more efficient resource use. By facilitating collaboration and delivering precise, data-driven insights, AI empowers diverse healthcare teams to address the full spectrum of patient needs.

AI-based models for risk stratification and clinical outcome prediction are transforming the management of infectious diseases in older adults. These technologies not only improve predictive accuracy but also support the delivery of personalized, evidence-based care tailored to the complex needs of this vulnerable population. The integration of AI into clinical decision support systems and multidisciplinary care frameworks enhances coordination and efficiency. As AI technologies continue to evolve, they hold the potential to optimize clinical outcomes, streamline resource utilization, and elevate the overall quality of care for older adults facing infectious diseases.

## 6. Antimicrobial Therapy: Clinical Personalization and AI-Supported Stewardship

AI and ML applications have demonstrated considerable potential in enhancing the clinical personalization of antimicrobial therapy and strengthening antimicrobial stewardship (AMS), particularly relevant in older adult populations. ML models have been effectively employed to guide both empirical and targeted antimicrobial selection by integrating complex clinical and microbiological variables such as comorbidities, prior antibiotic exposure, local resistance patterns, organ function, comorbidities, and real-time Electronic Health Record (HER) data. For instance, a deep learning-based optical system was developed to automate and accelerate antimicrobial susceptibility testing [60]. The model achieved high discriminative performance (mean AUC: 0.87).

Retrospective case studies reinforce the clinical applicability of these tools. In the United States, the PyTorch EHR deep learning model achieved an AUC of 0.911 for predicting Methicillin-Resistant Staphylococcus aureus (MRSA) positivity using time-series EHR data, outperforming traditional models and enhancing early therapeutic decision-making [61]. Similarly, in ICU settings, random forest classifiers have been used to predict the risk of carbapenem-resistant Gram-negative infections, demonstrating improved accuracy (84%) and enabling timely interventions [62].

Moreover, ML algorithms have been used to calculate optimal dosing strategies in the presence of renal or hepatic dysfunctions, especially renal or hepatic impairment, addressing a common clinical need in geriatric care [63]. In surgical settings, AI models assessing prophylactic adequacy have yielded outstanding predictive performance (AUC > 0.97), supporting context-specific antibiotic management [64]. At the institutional level, AI has also been applied to detect inappropriate prescribing patterns and to promote AMS. A recent systematic review evaluating AI applications in antimicrobial stewardship programs found that ML models not only outperformed traditional rule-based approaches in sensitivity and negative predictive value but also provided explainable support for early detection of inappropriate prescriptions and multidrug-resistant organisms, particularly in vulnerable populations such as older adults [65]. For instance, decision-tree-based models have identified misuse of agents such as piperacillin/tazobactam, offering real-time feedback for quality improvement initiatives and educational interventions [66]. Importantly, AI-driven systems have supported prospective audit-and-feedback mechanisms by offering interpretable predictions to clinicians, thereby facilitating acceptance and appropriate use of AI recommendations. These tools also aid in early identification of candidates for de-escalation, reducing antimicrobial pressure and supporting resistance prevention strategies [67].

A recent meta-analysis confirms the high diagnostic and predictive performance of AI tools across AMS settings, with pooled AUC, accuracy, and sensitivity estimates ranging from 72% to 77%, and negative predictive values approaching 80% [68]. Notably, these tools can enhance safety and efficacy in the geriatric population, which is disproportionately affected by adverse drug reactions and multidrug-resistant infections [69]. In this perspective, AI-enabled antimicrobial therapy represents a major step toward precision medicine, enhances therapeutic safety, reduces drug-related complications, and promotes sustainability in older adults [70,71]. By optimizing both patient-level and institution-wide prescriptions, these tools support therapeutic safety, reduce unnecessary antibiotic exposure, and foster sustainable prescribing practices aligned with the complex needs of ageing populations.

## 7. Prevention, Surveillance and Control of Healthcare Associated Infections (HAIs)

In older adult populations, AI is emerging as a pivotal tool for the prevention and control of HAIs, through early risk stratification, outbreak surveillance, and the implementation of targeted interventions. ML models can analyze EHRs and device-derived data to identify high-risk elderly patients, such as those with indwelling catheters, mechanical ventilation, or prosthetic implants, who are particularly susceptible to HAIs. This enables the timely application of preventive measures, including the reinforcement of hygiene protocols or the early removal of invasive devices.

These predictive algorithms often demonstrate high levels of diagnostic accuracy. A recent meta-analysis reported a pooled sensitivity of approximately 84%, a specificity of 90%, and an AUC of around 0.86 for AI-based models in HAI surveillance, outperforming or matching the accuracy of conventional manual infection tracking [72]. Specifically, models addressing the most common HAIs, such as urinary tract infections and surgical site infections, frequently exceed an AUC of 0.80, underscoring their robust reliability [73].

AI-powered surveillance systems, implemented within hospitals and long-term care facilities, can continuously monitor real-time clinical signals to detect outbreaks earlier than traditional methods, triggering timely alerts in geriatric wards or residential care settings. For instance, an AI-driven monitoring platform deployed in nursing homes was able to flag early signs of respiratory or urinary infections 2 to 4 days prior to clinical diagnosis (AUC = 0.86) [74]. Likewise, an automated syndromic surveillance network across elder care facilities demonstrated earlier outbreak detection and reduced outbreak magnitude compared to conventional reporting systems [75].

Predictive analytics also inform tailored preventive actions: by identifying which patients or environments carry the highest risk, AI can assist infection control teams in deploying focused interventions, such as targeted decolonization, optimized sanitation schedules, or preemptive isolation, to contain environmental reservoirs of pathogens. In practice, these tools support epidemiological management by synthesizing complex data into actionable insights, enabling infection prevention specialists to allocate resources efficiently and respond swiftly in high-risk geriatric settings [76].

Initial implementations have shown promising outcomes: one hospital reported an 85% reduction in manual surveillance workload and a halving of HAI incidence following the deployment of an AI-assisted surveillance system [28]. Although routine clinical adoption remains in its early stages due to challenges related to system integration and workflow alignment, current evidence strongly suggests that AI can significantly enhance the prevention, monitoring, and control of HAIs in geriatric care, offering superior accuracy, timeliness, and adaptability when compared to traditional approaches.

## 8. Continuity of Care and Reduction in Readmissions

**Figure 4:**
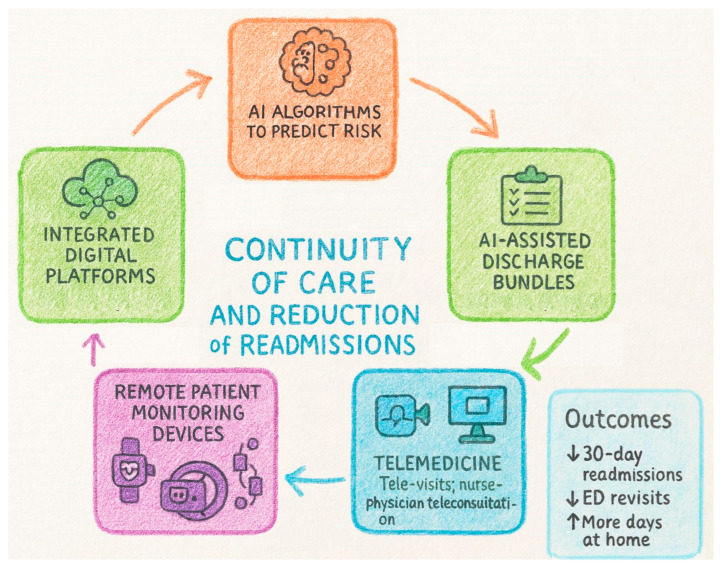
Framework showing how AI and digital health tools support continuity of care and reduce readmissions through risk prediction, assisted discharge, telemedicine, remote monitoring, and integrated platforms, improving post-discharge outcomes. Arrows: ↓ decrease; ↑ increase. Abbreviations: AI, artificial intelligence; ED, emergency department.

Older adults recovering from infection are vulnerable to early deterioration and unplanned rehospitalization because of frailty, multimorbidity, polypharmacy, cognitive impairment, and atypical recovery patterns. Aligning continuity-of-care tools with these geriatric constructs is essential for safe discharge and sustained recovery (Figure 4).

### 8.1. AI Algorithms to Predict Risk of Post-Infection Readmission

Post-sepsis and post-pneumonia readmissions remain frequent and costly; ML models using EHR and claims data can identify patients at high risk within 30 days of discharge [77]. In multicenter evaluations of AI-enabled sepsis prediction embedded in workflow, implementation was associated with reductions in in-hospital mortality, length of stay, and 22.7% lower 30-day readmission, suggesting a downstream impact on continuity outcomes [78]. ML models tailored to older adults and multimorbidity are emerging: recent geriatric readmission models and ML frameworks have shown improved discrimination over traditional scores, focusing on social and functional determinants. For pneumonia, national-database analyses demonstrate that gradient-boosting and rule-based models identify comorbidity, illness severity, discharge disposition, and payer type as key drivers [79]. High-performing models combine structured data with unstructured notes via natural language processing to evaluate geriatric complexity, functional status and social risk. This, coupled with age- or frailty-calibrated thresholds, enables earlier identification of frail patients needing intensified follow-up and risk-tiered actions, such as pharmacist review for polypharmacy, early home visits, telemonitoring enrolment, etc. [77]. Implemented in this way, AI-guided, risk-stratified transitional care is associated with measurable reductions in 30-day post-infection readmissions in older adults.

### 8.2. Planning Assisted Discharge and Home Patient Monitoring

Assisted discharge bundles (discharge reconciliation, patient/caregiver teaching, and scheduled follow-up) reduce readmissions in older adults; augmenting transitional-care programs with AI-derived risk and needs assessments further decreases rehospitalization [80]. Systematic evidence indicates that EHR-based interventions (alerts, care-plan automation, post-discharge tasking) are associated with 17–28% reductions in 30-/90-day readmissions across RCTs [81]. Discharge planning tools should: (i) embed AI risk at the point of discharge, (ii) auto-generate individualized follow-up (infectious-disease reviews, renal labs for nephrotoxic agents, vaccine appointments) [82], (iii) flag red-flag symptoms tailored to atypical presentations (e.g., delirium, falls), and (iv) route tasks to community nurses and caregivers.

Home patient monitoring after infectious discharge relies on simple vital-sign kits and symptom diaries. Often those records are not integrated with the EHR, and they are corrected and analyzed in a comprehensive way only during hospital readmission [83]. Implementing AI-driven trend detection and more frequent home visits allow the interpretation of early relapses (tachycardia, hypoxia, fever) and medication-related toxicity.

### 8.3. Integration with Telemedicine, Wearable Devices, and Digital Platforms

Remote patient monitoring (RPM) and telehealth can detect deterioration early, but effects on readmissions vary by condition and program design. Systematic reviews in hospital-at-home and post-acute populations highlight feasibility, early detection, and heterogeneity of impact [84]. Telemonitoring reduces readmissions in chronic obstructive pulmonary disease (COPD) and pneumonia, with less consistent benefit in heart failure [85,86]. Telemedicine can extend post-infection follow-up for older adults by combining scheduled tele-visits with real-time teleconsultation between home-visiting nurses and supervising physicians. In Hospital-at-Home programs, remote visits were non-inferior to in-home visits for safety and experience [87]. 

Remote patient monitoring ranges from non-wearable home kits (pulse oximetry, Blood Pressure, temperature) to wearables that stream physiology (heart rate, respiratory rate, nocturnal SpO_2_, activity) [88]. During and beyond COVID-19, home oximetry programs proved feasible and generally safe [89]. Wearables for older adults are increasingly studied: reviews document usability considerations and sensor choices relevant to detecting infectious relapse [90]. 

Digital care platforms can close the hospital-to-community gap by unifying tele-visits, RPM feeds, medication surveillance, and shared care plans across hospital, primary care, and community nursing. World Health Organization (WHO) guidance emphasizes that digital interventions should strengthen, not replace, care systems, with attention to feasibility and equity [91].

### 8.4. Support for Integrated Territorial Care Models and Safe Hospital-to-Community Transition

Integrated territorial care aligns with WHO’s Integrated Care for Older People (ICOPE) approach, emphasizing person-centered assessment, shared care plans, and community follow-up, now supported by digital tools and decision support [92]. AI and IoT-enabled transitional pathways can coordinate primary care, home nursing, and social services, with early pilot evidence of feasibility and proposed outcome frameworks [88,93].

## 9. Current Limitations and Prospects

The application of AI in the geriatric infectious disease domain offers both substantial promise and notable limitations. On the one hand, ML and deep learning algorithms have demonstrated encouraging capabilities in enhancing diagnostic accuracy, prognostic stratification, and the personalization of therapeutic strategies for older adults, for instance, by optimizing antimicrobial stewardship and enabling the early identification of patients at risk for infectious complications. On the other hand, the current literature underscores that evidence specifically tailored to the geriatric population remains limited and fragmented.

**Figure 5:**
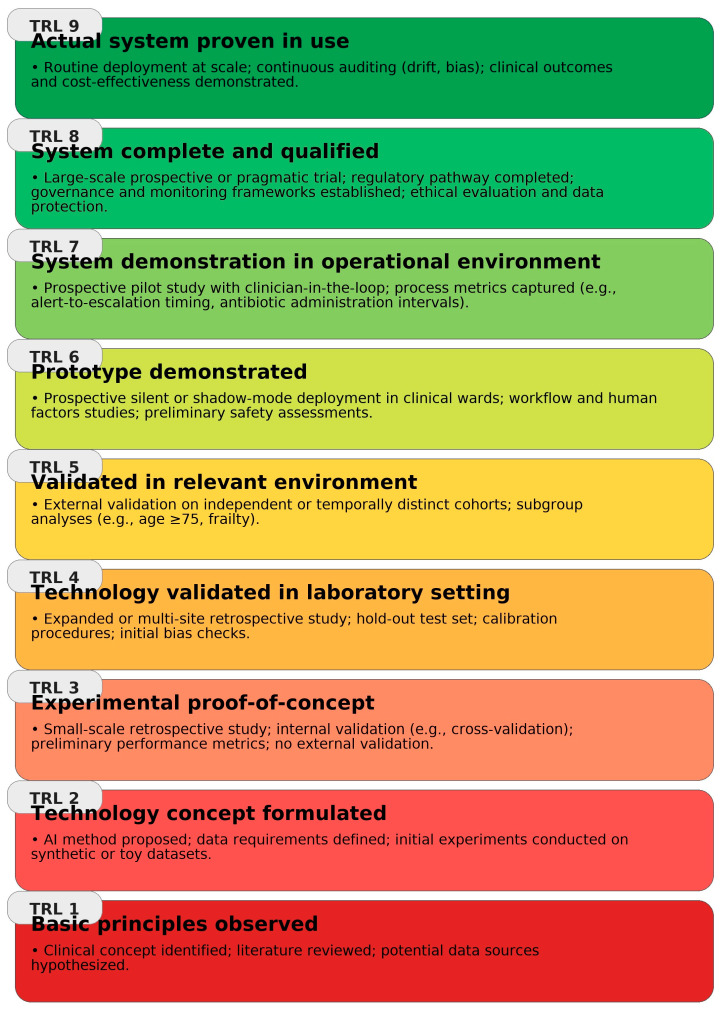
Clinical AI readiness for infection management in older adults depicted using the Technology Readiness Levels (TRL 1–9) scale, adapted from NASA’s TRL framework; each level summarizes the expected evidence and setting, from basic principles/concept (TRL-1) to routine deployment with continuous auditing and demonstrated clinical and cost-effectiveness (TRL-9).

Building on NASA’s Technology Readiness Level (TRL) framework [94], Figure 5 synthesizes the implementation maturity of clinical AI use-cases across the geriatric infection pathway, highlighting domains with stronger external/prospective evidence versus those where deployment remains preliminary.

To better delineate the scope and maturity of the current body of evidence, Table 1 systematically synthesizes the principal study designs, target populations, evaluated endpoints, and reported methodological limitations. This tabular overview complements the qualitative synthesis by providing a concise framework for assessing both the strengths and the translational gaps of AI applications in geriatric infectious disease care.

### 9.1. Limits of Current Evidence and Applications

Despite the growing body of research on AI in medicine, there remains a striking paucity of studies specifically targeting older adults, leading to the underrepresentation of geriatric patients in the training datasets of AI models. This demographic gap can introduce systematic biases and compromise the generalizability of algorithms to frail populations, thereby undermining both the equity and reliability of AI-driven clinical decisions [96]. In particular, the underrepresentation of older and frail adults in AI training datasets represents a critical source of bias, as models trained primarily on younger or healthier populations may fail to capture the complexity and heterogeneity of geriatric patients. Indeed, geriatricians and data scientists have warned that AI may inadvertently exacerbate health disparities in geriatric care due to biased model development and the lack of external validation on appropriately aged populations [97]. This limitation can reduce model accuracy, compromise external validity, and risk exacerbating health disparities. To date, only a handful of prospective multicenter studies have evaluated the impact of AI systems on clinically meaningful outcomes in older adults, and the absence of such rigorous trials continues to cast uncertainty on the true clinical benefit and cost-effectiveness of these tools in geriatric practice. Future research should therefore prioritize the development of dedicated, representative datasets and multicenter, externally validated studies that include sufficient numbers of frail and very old individuals to ensure equitable and generalizable AI applications in geriatric infectious disease care.

Another limitation concerns the methodological rigor underlying the reported high performance of many AI models. AUC values approaching 0.98, while impressive, may conceal overfitting when derived from retrospective analyses on restricted or homogeneous datasets. Few studies explicitly report how missing data, variable selection, or hyperparameter tuning were handled, limiting the assessment of model robustness and reproducibility. Furthermore, calibration analyses, essential to determine whether predicted risks align with observed outcomes, are rarely presented, making it difficult to evaluate clinical reliability beyond discrimination metrics. The lack of standardized benchmarking against optimally specified conventional statistical models also risks inflating the apparent superiority of AI approaches. Importantly, the gap between algorithmic performance in controlled research settings and effectiveness in routine geriatric care remains wide: most systems have not been tested prospectively within clinical workflows, where multimorbidity, frailty, and heterogeneous data quality may markedly degrade performance. Bridging this translational gap will require prospective studies embedded in real-world care pathways, systematic calibration and reproducibility assessments, and the inclusion of clinically meaningful endpoints such as treatment adequacy, timeliness of intervention, and patient-centered outcomes.

Beyond high-level considerations, three practical domains shape adoption in geriatric settings: (i) data quality and interoperability: heterogeneous EHR schemas, non-random missingness, and site-to-site microbiology variability limit generalizability; multi-institutional or federated data and standardized pipelines can mitigate this [98]; (ii) prospective, externally validated evidence: most studies remain retrospective/single-center; few pilots or pragmatic trials report calibration and fairness auditing, constraining real-world reliability [99]; (iii) organizational readiness and workflow fit: limited leadership support, and insufficient clinician training hinder EHR integration and sustained use; explainability is essential to clinician trust and to avoid alert fatigue. Targeted enablers include governance for privacy/ethics, interpretable models with routine calibration (e.g., SHapley Additive exPlanations SHAP/Local Interpretable Model-agnostic Explanations LIME, calibration plots), EHR-native deployment, and structured education [100].

An additional limitation concerns the practical implementation of AI across various geriatric care settings. Long-term care facilities and community-based home care services frequently suffer from low levels of digitalization and poorly integrated health information infrastructures, posing significant barriers to the deployment of advanced data-driven tools. Even in hospital environments, integrating AI algorithms into existing clinical workflows can encounter organizational and resource-related obstacles, particularly in the absence of dedicated managerial support [101]. Furthermore, current AI models are often opaque and difficult to interpret, so-called “black boxes”, which hinders their usability in everyday geriatric clinical practice [102]. Clinicians often express understandable reluctance to rely on recommendations generated by non-explainable algorithms, fearing unforeseen errors and challenges in reconciling such outputs with their clinical judgment. The lack of interpretable and transparent models thus hampers trust among healthcare professionals and constitutes a major barrier to the widespread adoption of AI in the care of frail older adults [103]. Furthermore, many of the available studies are limited by small sample sizes, single-center settings, and restricted inclusion of very old or frail patients, which further constrains the generalizability of their findings. Lastly, the included studies are highly heterogeneous in terms of objectives, design, populations, and analytical approaches, which limits direct comparability and makes it difficult to draw definitive conclusions. Future research should therefore prioritize well-designed, multicenter, and externally validated studies that adhere to rigorous scientific methodology to ensure reproducible and generalizable results in geriatric infectious disease care.

### 9.2. Development Perspectives

Considering these limitations, several avenues for development have emerged to ensure that AI becomes a truly effective and safe tool for the management of infections in geriatric settings. Foremost among these is the urgent need to build dedicated datasets for the frail elderly population, enriching AI models with specific data (clinical, laboratory, and functional) pertinent to individuals aged 75 and older. Targeted data collection initiatives, ideally supported by multicenter consortia and geriatric biobanks, may enhance representativeness and mitigate age-related biases in algorithmic performance.

Concurrently, the co-design of AI tools in close collaboration with multidisciplinary teams, including geriatricians, infectious disease specialists, pharmacologists, nurses, and data scientists, is essential to ensure that the solutions developed address real-world clinical needs. Such participatory approaches foster the creation of models attuned to the geriatric context, accounting for factors such as multimorbidity, polypharmacy, and frailty, while also facilitating user acceptance from the early stages of development [104]. In the broader context of combating antimicrobial resistance, it has likewise been emphasized that research must be steered toward more interpretable AI models and that interdisciplinary collaboration is key to integrating these technologies equitably and sustainably into care pathways [95].

Another critical perspective involves the integration of AI into regional and national health information systems. This requires the development of interoperable infrastructures that allow algorithms to access electronic health records, public health registries, and other relevant data sources securely, while fully complying with privacy regulations. Investing in advanced digital platforms within long-term care facilities and community healthcare settings will be instrumental in bridging the digital divide, thereby ensuring that AI-based surveillance and decision support tools can also be deployed in resource-limited or peripheral contexts [105].

Simultaneously, the development of “explainable” AI models, endowed with transparent and interpretable decision-making logic, will be imperative. The adoption of principles from Explainable AI (XAI) and ethics-by-design can help overcome the “black box” problem, enhancing the perceived trustworthiness of these technologies. The ability to trace and communicate the rationale behind a prediction (e.g., using techniques such as LIME or SHAP) is, in fact, crucial for fostering clinician and patient acceptance of algorithmic support.

Finally, greater emphasis must be placed on the training of healthcare professionals in the critical use of AI. Physicians, nurses, and other practitioners will need to acquire AI literacy and develop the competencies necessary to interpret algorithmic recommendations appropriately and integrate them into clinical decision-making. A recent systematic review on the subject underscored that clinician education, coupled with the implementation of XAI techniques, constitutes a foundational step toward improving both understanding and acceptance of artificial intelligence in routine practice [106].

From an implementation standpoint, workflow integration requires EHR-native deployment with outputs available early in the admission episode; Ali et al. [107] illustrate ML models trained on admission clinical data with SHAP explanation, an approach conducive to timely decision-making. Training should build AI literacy around feature-attribution to support clinician acceptance and safe escalation pathways. Cost-effectiveness ultimately requires prospective/pragmatic evaluations linking model-driven actions to resource use and outcomes. Ethical/legal safeguards entail Institutional Review Board (IRB) oversight and robust data-protection/governance across sites, acknowledging missingness and documentation variability that affect reliability and accountability in AI-supported decisions [107].

Taken together, these measures, when guided by clear ethical and governance frameworks, will lay the groundwork for the effective, safe, and equitable implementation of AI in infectious disease management for older adults, ultimately advancing a model of care that is both data-driven and profoundly person-centered.

## 10. Conclusions

AI is increasingly emerging as a high-potential resource to enhance diagnostic accuracy, refine prognostic stratification, and personalize the therapeutic management of infections in older adults, thus addressing the growing clinical complexity of the geriatric population. However, for these tools to yield tangible and measurable benefits, rigorous validation, seamless integration into care pathways, and careful adaptation to the infectious and pathophysiological specificities of advanced age are indispensable.

This trajectory demands a structured alliance of expertise: only through the synergistic collaboration of clinicians, data scientists, epidemiologists, public health professionals, and institutional decision-makers can implementation be rendered effective, safe, and equitable. The future of geriatric infectious disease medicine will thus be increasingly data-driven and AI-supported, yet also deeply centered on the individual, on their clinical history, personal values, and inherent vulnerabilities.

Technological progress must be guided by an ethics of care: the algorithm must remain in service of the person, and not the other way around. Ultimately, the future of geriatric infectiology will be shaped by powerful algorithms—but it must always speak the language of human frailty.

## Figures and Tables

**Table 1:** Summary of study types, AI models, data types, performance metrics, populations, endpoints, and limitations across the included literature on AI applications in geriatric infectious disease care.

Study Type	AI Models	Data Types	Performance Metrics	Populations	Endpoints	Limitations
Diagnostic studies [30,31,36,41,44,45]	Random forest, deep learning (CNN, RNN), NLP	EHR, clinical notes, laboratory tests, imaging	AUC up to 0.98; sensitivity 0.80–0.92; specificity 0.75–0.89	Older adults with suspected infections (sepsis, pneumonia), hospital and ICU settings	Diagnostic accuracy; early detection of infection	High AUCs from retrospective single-center datasets; predominant reliance on AUC without calibration; risk of overfitting; limited external validation
Prognostic / risk stratification models [3,53,54]	Gradient boosting, random forest, logistic regression, deep learning	Demographics, comorbidities, vital signs, labs, frailty indices	AUC 0.75–0.92; calibration rarely reported	Older inpatients with infections; ICU and emergency settings; multimorbid geriatric cohorts	Prediction of sepsis, septic shock, mortality, complications, frailty-related outcomes	Scarce calibration reporting; heterogeneous predictors; small geriatric-specific samples; few prospective trials; lack of standardized benchmarking vs. conventional models
Antimicrobial stewardship applications [65,66,68,95]	Rule-based ML, ensemble models, deep learning	Microbiology data, antibiograms, EHR	AUC 0.70–0.85; accuracy 0.75–0.80	Elderly inpatients, ICU patients, surgical populations	Antibiotic selection, resistance prediction, dosing strategies	Opaque models; heterogeneous variables; limited integration into stewardship workflows; little reporting on effectiveness in routine care
HAI prevention and surveillance tools [5,28,72,73]	NLP, decision trees, logistic regression	EHR, infection surveillance data, clinical notes	AUC 0.80–0.86	Elderly residents in hospitals, nursing homes, long-term care facilities	Detection of HAIs, outbreak identification, reduction in surveillance workload	Data heterogeneity; limited real-world deployment; reliance on retrospective validation; absence of systematic cost-effectiveness assessments
Continuity of care / readmission prediction [77,78,81,85,86]	Random forest, gradient boosting, deep learning	EHR, discharge summaries, claims data	AUC 0.70–0.82;	Older adults post-discharge after sepsis, pneumonia, or other infections	30-day readmission risk, mortality, functional decline, care coordination	Few multicenter prospective evaluations; limited frailty-specific adaptation; infrastructural barriers; lack of validation within learning health system frameworks

AUC: area under the curve; AI: artificial intelligence; CNN: convolutional neural network; EHR: electronic health record; HAI: healthcare-associated infection; ICU: intensive care unit; ML: machine learning; NLP: natural language processing; RNN: recurrent neural network; vs.: versus.

## Data Availability

No new data were created or analyzed in this study. Data sharing is not applicable to this article.
